# Survival and long-term surgical outcomes after colorectal surgery: are there any gender-related differences?

**DOI:** 10.1007/s13304-022-01323-4

**Published:** 2022-07-09

**Authors:** Pasquale Losurdo, Manuela Mastronardi, Nicolò de Manzini, Marina Bortul

**Affiliations:** Surgical Clinic Unit, Division of General Surgery, Department of Medical and Surgical Sciences, Hospital of Cattinara, University of Trieste, Strada di Fiume 447, 34149 Trieste, Italy

**Keywords:** Colorectal cancer, Colorectal surgery, Gender differences, Prevention, Survival

## Abstract

Colorectal cancer (CRC) incidence and mortality seems to be lower in women than in men. The present study aims to evaluate the impact of gender on CRC diagnosis, treatment, and survival. This is a retrospective cohort study based on a single-center dataset of CRC patients from the University Hospital of Trieste (Italy). Data of 1796 consecutive CRC patients referred to our center from November 11th, 2004, to December 31st, 2017, were analyzed. Right-sided carcinomas are more frequent in women than in men; furthermore, women had a lower surgical complication rate. Men showed a higher 5- and 10-year mortality. This survival benefit for women was observed independently of the tumor localization. The 5-year hazard ratio (HR) for women vs men was 0.776 (*p* 0.003), and after 10-year 0.816 (*p* 0.017). Regarding the disease-free survival (DFS), 5 and 10-year HR was 0.759 (*p* 0.034) and 0.788 (*p* 0.07), respectively. On multivariable analysis, respecting tumor localization, the odds of female gender were higher than man with right colon disease. Male gender was more independently associated with age at the surgery time. Women survival advantage was higher than men, except for patients older than 80. Surgical outcome and survival after CRC surgical treatment seem to be gender related. For this reason, gender could play an important role in CRC diagnosis and therapy, allowing an earlier diagnosis in women.

## Introduction

Colorectal cancer (CRC) is one of the most frequent causes of cancer-related death [1]. The implementation of nationwide screening coloscopies may allow a decline in CRC mortality and surgical complication rate [2, 3]. In general, CRC incidence and mortality seems to be lower for women than for man [4, 5], with also a higher survival rate [6]. Nevertheless, even if the literature shows that gender is the single significant predictor of the relative advantage of survival [6, 7], several pre-clinical and clinical studies do not focus on it [8], and female participants rate assesses around 38.8% [9]. Moreover, hormone balance may be an influencing factor with therapeutic potential [10]. Strikingly, right-sided colon cancer is more frequent in women than in men, presenting with a more aggressive form [4].

Considering that gender can strongly influence CRC diagnosis, treatment, and survival, women underrepresentation should be considered a strong bias, resulting in a validity impairment of those studies.

In the present study, we aim to evaluate the impact of gender on CRC diagnosis, treatment, and survival.

## Methods

### Study population

This is a monocentric retrospective cohort study on prospectively collected medical records of CRC patients from the University Hospital of Trieste (Italy).

Every patient signed an informed consent for data unspecified use and the local ethical committee approved the anonymously data use, according to our legislation (GDPR 679/2016, Par.26) [11].

We considered a consecutive series of 1796 patients diagnosed with CRC who referred to our center between November 11, 2004, and December 31, 2017. A total of 193 patients (10.7%) were excluded due to the incomplete data. Individual information was collected from the patients or the clinical hospital software.

All included patients underwent cancer surgical resection. The following data were analyzed: age at diagnosis, CEA and CA 19.9 levels, TNM classification, the American Joint Committee on Cancer (AJCC) stage, elective and emergency surgery rate, kind of surgery performed (i.e., right, left, or transverse colectomy, or anterior rectal resection), American Society of Anesthesiologists (ASA) score, operative time, length of hospital stay (LOS), total lymph node removed number, positive lymph node removed number, complications as splenic lesion, surgical site infection (SSI), anastomotic leakage (AL), and follow-up data as recurrences, life status, and mortality.

Anastomotic leakage is defined as any loss of the anastomosis confirmed by radiographic examination with fluid/air bubbles surrounding the anastomosis, extravasation of endoluminally contrast enema, and/or abscess at the level of anastomosis.

According to the impact on clinical management, the severity of anastomotic leakage is graded based on the classification of the “International Study Group of Rectal Cancer” as: Grade A (AL results in no change in patients management), Grade B (leakages manageable without re-operation), and Grade C (anastomotic leakage requires re-operation).

### Statistical analysis

Summary statistics of clinical and instrumental variables at enrolment were summarized by means of ± standard deviation (SD) or median and interquartile ranges (IQR), for continuous variables, while categorical variables are expressed as absolute and percentage frequencies.

The Kolmogorov–Smirnov normality test was used to identify data normal distribution. Continuous normal variables were compared with the unpaired student *t* test. Otherwise, the nonparametric Mann–Whitney *U* test was used. Nominal and ordinal variables were compared with the Chi-square test.

The Kaplan–Meier method was adopted to calculate the overall survival (OS) and the Disease-free survival (DFS) from the diagnosis date to the date of death or the last follow-up visit.

**Fig. 1:**
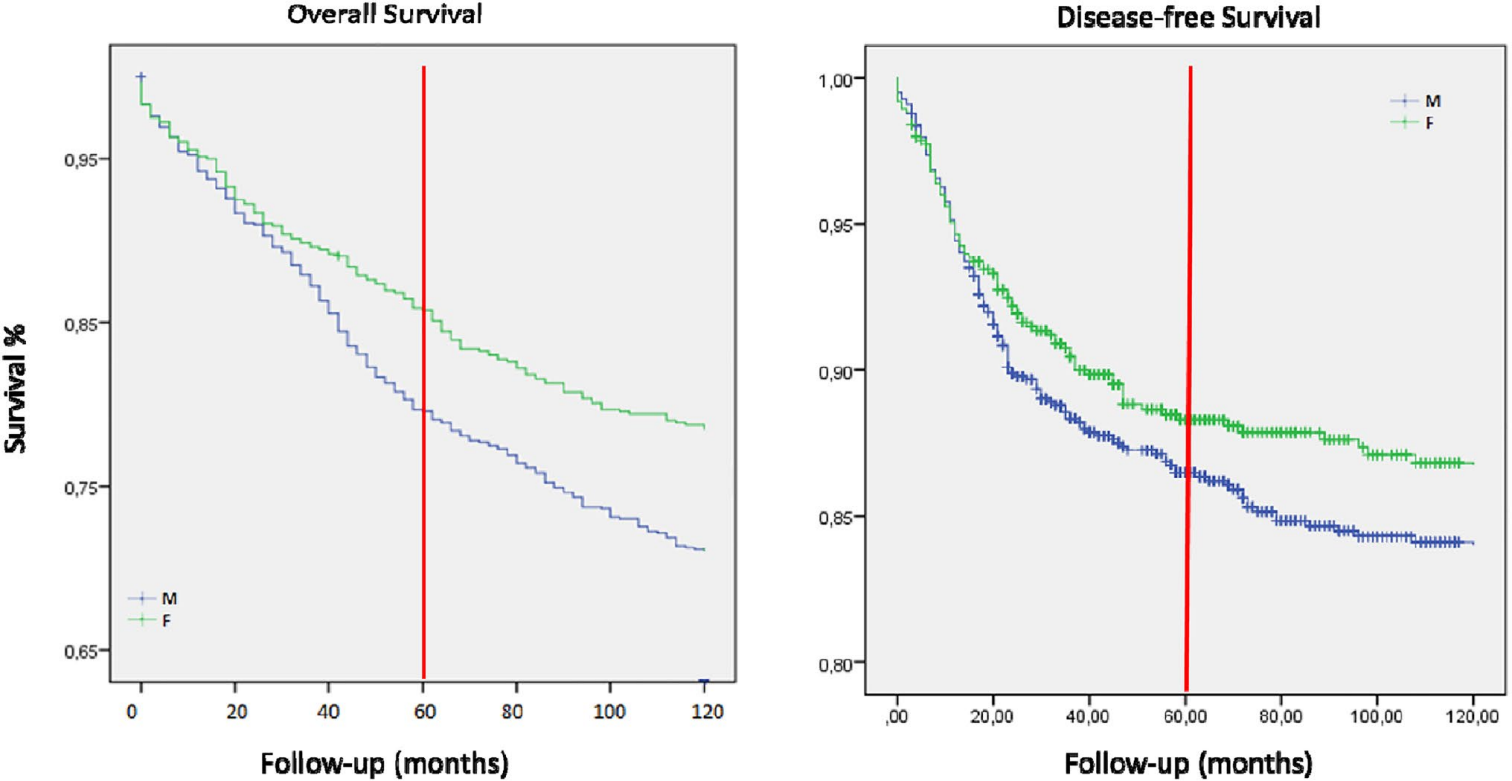
Kaplan–Meier Survival Curves: gender-related OS and DFS (Red line refers to 5-year follow-up).

To estimate disease-free survival (DFS), we consider the date of diagnosis till the date of the “event”, defined as the first occurrence of locoregional relapses. The presence of distant metastases was considered as an additional event. Patients without recurrence were considered till the date of last follow-up or death. A *p* value from the log-rank test of 0.05 or less was considered significant.

To estimate the association between impact of gender and potential predictors, univariate cox regression models were estimated for each parameter statistically significant at the descriptive analysis.

Statistically significant variables at univariable analyses were selected as candidate prognostic factors and incorporated into multivariable logistic regression models.

All significance tests were two-sided with a significance level of 0.05; the results are displayed as *p* values or 95% confidence intervals (CI).

Statistics were performed using IBM SPSS 25.0 (IBM Corp., Armonk, New York, USA) and the R package version 3.10.

## Results

Demographic, clinical, and histopathological information according to gender is shown in Table 1.

**Table 1:** Main clinical characteristics of the patients included in the study

	**Overall** *N* = 1603	**M** *N* = 843 (52.6%)	**F** *N* = 760 (47.4%)	*p* value
Age	71 ± 12	71 ± 10	72 ± 11	–
< 50 yo	54 (%)	25 (2.98%)	29 (3.8%)	–
51–60 yo	202 (%)	110 (13.1%)	92 (12.1%)	–
61–70 yo*	423 (%)	244 (29.1%)	179 (23.6%)	0.05
71–99 yo*	917 (%)	464 (55.5%)	453 (59.6%)	0.02
CEA	33 ± 300 3 (IQR 0–4995)	30.1 ± 283 2.86 (0–4995)	37.4 ± 325 2.78 (0–4670)	–
CA19-9	669 ± 526 10 (IQR 1–9500)	81 ± 635 9.52 (IQR 1–9500)	46 ± 296 9.90 (IQR 1–4264)	–
ASA 1–2*	817 (50.9%)	407 (48.3%)	410 (53.9%)	0.019
ASA 3–4	561 (35%)	330 (39.2%)	231 (30.4%)	
Elective surgery	1397 (87.1%)	738 (87.6%)	659 (86.7%)	–
Emergency surgery	206 (12.9%)	105 (12.4%)	101 (13.3%)	–
Right colectomy*	547 (33.7%)	261 (31%)	286 (37.6%)	0.05
Transverse colectomy	89 (5.6%)	49(5.8%)	40 (5.3%)	–
Left colectomy	138 (8.6%)	73 (8.7)	65 (8.6%)	–
ARR*	818 (51%)	454 (53.9%)	364 (47.9%)	0.03
Operative time*	177.5 ± 69	186 ± 72	165 ± 62	0.0001
LOS	12 ± 14	12 ± 16	11 ± 10	–
Total lymph node*	17 ± 10 15 (IQR 5–31)	17 ± 10	18 ± 10	0.003
Positive lymph node*	1 (IQR 0–4)	1 [IQR 0–3]	2[IQR 0–4]	0.037
Type of surgical complications				
Splenic lesion	9 (0.6%)	8 (0.8%)	1 (0.1%)	–
SSI	121 (7.5%)	73 (7.2%)	48 (6.3%)	–
AL*	185 (11.5%)	118 (11.6)	67 (8.8%)	0.04
AL type B–C*	98 (6.1%)	64 (6.3%)	34 (4.5%)	0.01
AL type A*	87 (5.4%)	54 (5.3%)	33 (4.3%)	0.06
Complication rate according to Clavien-Dindo classification				
1–2	1303 (81.3%)	747 (69.4%)	556 (73.2%)	0.044
3–5	184 (11.5%)	125 (12.5%)	59 (9%)	
TNM				
pTis	69 (4.3%)	35 (4.2%)	34 (4.5%)	–
pT1-2	489 (30.1%)	254 (30.1%)	235 (30.9%)	
pT3-4	765 (47.4%)	424 (50.3%)	341 (44.7%)	
pN +	471 (29.4%)	255 (30.2%)	216 (28.3%)	–
pM*	143 (8.9%)	71 (8.4%)	72 (9.4%)	0.025
AJCC stage				
I–II	821 (51.2%)	435 (51.6%)	386 (50.6%)	–
III–IV	782 (48.8%)	408 (48.3%)	374 (49.2%)	

Values are mean ± SD, %, or median [interquartile range]

*AL* anastomotic leakage, *ARR* anterior rectal resection, *ASA* American society of anesthesiologists score, *AJCC* American joint committee on cancer, *CA* 19-9 carbohydrate antigen 19-9, *CEA* carcino-embryonic antigen, *F* female, *LOS* length of hospital stay, *M* male, *SD* standard deviation, *SSI* surgical site infection, *yo* years old

*Statistically significant difference between men and women

A total of 1603 CRC patients, divided into 760 female (47.4%, F) and 843 male (52.6%, M), were included in the study.

According to gender, patients’ demographic, clinical, and histopathological characteristics were strictly homogeneous.

Globally, women were older than man at diagnosis time, but the majority of men were detected in the screening age; instead, women number with a primary diagnosis of cancer rapidly raise at the end of the screening time.

Colon sigmoid and rectum cancer were the most frequent in both women and men. However, women showed a higher prevalence of right-sided cancer (37.6% F vs. 31% M; *p* < 0.05).

Remarkably, man presented with more pathological advanced tumors (pT3-4) than women but with a not-significantly higher AJCC stage at primary CRC diagnosis.

### Surgical outcome

Most of the surgical procedure was performed in the elective setting. Women had a better clinical condition according to ASA score (ASA score 1–2: 48.3% M vs 53.9% F; *p* 0.019) and less surgical complication according to Clavien-Dindo classification (Clavien-Dindo 3–5: 12.5% M vs 9% F, *p* 0.044).

Anastomotic leakage (AL) was significatively high in men (11.6 vs 8.8%; *p* 0.04). Remarkably, AL was equally divided into type a and type b-c fistula in woman. Nevertheless, type b-c fistulae were more frequent in men.

A higher number of lymph nodes were removed in women (18 ± 10 F vs. 17 ± 10 M; *p*  0.003), also showing a higher lymph node ratio (number of positive lymph nodes/number of removed lymph nodes; *p* < 0.03).

### Survival

For the survival analysis, a median follow-up of 7.8 years was considered. Only 810 patients were analyzed for a 10-year follow-up. In our cohort, women showed both higher 5-year and 10-year survival (5-year OS: 80.5% M vs 86.9% F; *p* 0.724; 10-year OS: 73.3% M vs 80% F; *p*: 0.002; Fig. 1 and Table 2A). Better OS and DFS in women are even more prominent at 10 years (*p* 0.002 and *p* 0.03 respectively). Moreover, this survival benefit was observed even independently of tumor localization.

**Table 2:** (A) Gender-related 5- and 10-year overall and disease-free survival. (B)Univariate regression analysis for gender-related 5- and 10-year overall and disease-free survival

	5-year OS	*p* value	10-year OS	*p* value	5-year DFS	*p* value	10-year DFS	*p* value
**Male**	80.5%	0.724	73.3%	0.002	87.3%	0.565	84.4%	0.03
**Female**	86.9%		80%		89.1%		87.1%	

OS	HR	95% CI	*p* value	DFS	HR	95% CI	*p* value
**5-year OS**	0.776	0.657–0.916	0.003	5-year DFS	0.759	0.586–0.983	0.034
**10-year OS**	0.816	0.690–0.965	0.017	10-year DFS	0.788	0.607 – 1.022	0.07

*DFS* disease-free survival, *OS* overall survival

The univariate regression analysis showed slightly higher OS and DFS rates for women (Table 2B). The 5-year hazard ratio (HR) for women vs men was HR 0.776 (IC 95% [0.657–0.916]; *p* 0.003), and after 10-year HR was 0.816 (IC 95% [0.690–0.965]; *p* 0.017).

Regarding DFS, 5 and 10-year HR was 0,759 (IC 95% [0.586–0.983]; *p* value 0.034) and HR 0.788 (IC 95% [0.607–1.022]; *p* value 0.07), respectively.

On multivariable analysis (Table 3), with respect of tumor localization, the odds of female gender were higher among man with right colon disease (odds ratio (OR) 9.172; 95% CI 1.717–19.8; *p* 0.036), whereas male had significantly higher odds of having a left colon disease (OR 5.75; 95% CI 2.57–10.23; *p* < 0.001).

**Table 3:** Multivariate independent predictors of survival

Variables	Male gender			Female gender		
	OR	CI 95%	*p* value	OR	CI 95%	*p* value
< 50 yo	0.897	0.801–1.014	0.065	0.701	0.65–1.004	0.121
61–70 yo	2.20	1.02–4.75	0.044	1.12	0.47–2.66	0.801
71–99 yo	2.93	1.79–5.56	0.023	3.80	1.35–6.63	0.003
Right colon	1.21	0.39–2.12	0.201	9.17	1.72–19.8	0.036
Left colon	5.75	2.57–10.23	< 0.001	4.75	0.80–7.02	0.05
ASA > 2	5.12	2.02–19.49	0.004	5.75	2.33–11.78	0.001
AJCC III–IV	2.96	0.951–4.98	0.000	2.83	0.75–5.89	0.001

*ASA* American society of anesthesiologists score, *AJCC* American joint committee on cancer, *CI* confidence interval, *OR* odds ratio, *yo* years old

Male gender was more independently associated with age at the time of surgery. Only after 80 years old, gender was not associated to the age (M: OR 1.017; 95% CI 1.003–1.030; *p* 0.322; F: OR 1.10; 95% CI 1.010–1.231; *p* < 0.222).

The survival advantage of women as opposed to men is more pronounced in younger patients than 80 years old (M: OR 1.017; 95% CI 1.003–1.030; *p* 0.322; F: OR 1.10; 95% CI 1.010–1.231; *p* < 0.222).

## Discussion

To the best of our knowledge, this is the first gender-focused analysis that evaluates the long-term OS and DFS and surgical outcome of CRC. Being CRC one of the most frequent causes of cancer death in women worldwide [1], our findings might be helpful to identify variables that could be potentially relevant for CRC screening, diagnosis, and treatment in women. They might benefit from preventive measures, including extended colonoscopy time and substitutive hormone therapies, regardless of the optimization of medical therapy and surgical strategy.

Several studies show gender-related differences even in CRC molecular biology [12–14]. It seems that women have greater proximal tumors, with MSI-H and BRAF mutation, suggesting their origin from sessile serrated polyps. Moreover, interval cancer appears to develop more frequent in women, highlighting the importance of a gender-based change in screening methodology [12, 15, 16].

The present retrospective cohort study shows several differences between men and women. Globally, women are older than man. Considering that the current CRC screening program in Italy collects people aged between 50 and 69, the majority of men were detected during screening age; instead, the number of women with a primary diagnosis of cancer rapidly raises at the end of that period. Screening plays a key role in early diagnosis and surgical treatment. In fact, a lower stage at diagnosis could imply a more conservative surgical strategy, with a lower complications rate [3]. Moreover, considering that women tend to have a smaller bowel diameter and a longer transversum colon, standard coloscopy devices are not always suitable for them [17]. This assumes a particular importance since, for unclear reasons, right-sided cancer is more frequent in women [6, 18, 19], as confirmed by our study, and tumor location has to be considered an independent prognostic factor [20, 21]. Even if women older than 75 years accept screening coloscopy less than male [4, 6, 12], they would need it later than 69 years, as 59.6% of women in our cohort were older than 70 years when CRC was diagnosed.

It is well known that right-sided colon cancer causes more unspecific symptoms, manifesting later than left-sided cancer. This might explain the higher presence of metastasis at diagnosis in women in our cohort.

Despite this, in our cohort women showed a significantly better OS and DFS than men, according to a recent meta-analysis by Yang et al. [7] demonstrating a longer overall survival for women.

Considering the older age and the higher number of right-sided cancers, other factors could be responsible for this effect. Different studies stated the importance of female hormones in protecting against CRC [22, 23]. Estrogens suppress tumor growth, and their receptors are lower expressed in malignant colon tissue [24]. Moreover, exogenous estrogen has been shown to decrease serum levels of insulin-like growth factor (IGF-1) [25] and bile acid production, considered CRC mitogens [12]. A recent study by Amitay et al. [26] showed that postmenopausal hormonal therapy was associated with CRC risk reduction, with no major differences among the genetic subtypes. However, they stated that, considering that the overall health risks exceeded benefits, it may not be recommended as a public health measure to lower CRC risk among women.

In the present study, we observed a survival benefit in the whole population of women, but most of them were older than 50 years. This implies that hormone condition should be considered as a strong influencing factor. For this reason, further investigations about gender role in CRC development are demanded, especially concerning molecular mechanisms. To reach this goal, both pre-clinical and clinical research should include the differentiation in male and female population.

An important limit of the study is its retrospective design. Strengths are the huge patients’ number and a long-time follow-up. Moreover, comparisons in this large cohort could lead statistically significant differences, even if their clinical relevance may be limited.

## Conclusion

Surgical outcome and survival after CRC surgical treatment seems to be gender related. Considering that several factors significantly differ between men and women, gender should play a relevant role in CRC diagnosis and therapy. Moreover, specific tools should be adopted to allow an earlier diagnosis in women.
